# Effect of implant design on primary stability using torque-time curves in artificial bone

**DOI:** 10.1186/s40729-015-0024-0

**Published:** 2015-08-08

**Authors:** Yoko Yamaguchi, Makoto Shiota, Motohiro Munakata, Shohei Kasugai, Masahiko Ozeki

**Affiliations:** 1 Department of Implant Dentistry, Showa University School of Dentistry, 2-1-1 Kitasenzoku Ota-ku, Tokyo, 145-8515 Japan; 2 Oral Implantology and Regenerative Dental Medicine, Department of Masticatory Function Rehabilitation, Division of Oral Health Sciences, Graduate School, Tokyo Medical and Dental University, 1-5-45 Yushima, Bunkyo-ku, Tokyo, 113-8510 Japan; 3 Oral Implantology Department of Prosthodontic Dentistry for Function of TMJ and Occlusion, Kanagawa Dental University, 82, Inaokachou, Yokosuka-shi, Kanagawa 238-8580 Japan

**Keywords:** Primary stability, Implant design, Bone density, Torque-time curve, Insertion torque, Removal torque

## Abstract

**Background:**

Primary stability following implant placement is essential for osseointegration and is affected by both implant design and bone density. The aim of this study was to compare the relationships between torque-time curves and implant designs in a poor bone quality model.

**Methods:**

Nine implant designs, with five implants in each category, were compared. A total of 90 implants (Straumann: Standard RN, Bone Level RC, Tapered Effect RN; Nobel Biocare: Brånemark MKIII, MKIV) were placed in type IV artificial bone. Torque-time curves of insertion and removal were recorded at the rate of 1000 samples/s by a torque analyzer.

**Results:**

The torque-time curves were divided into initial, parallel, tapered, and platform areas. The mean torque rise rate of the parallel area was smallest at 0.36 N · cm/s, with a significant difference from those of the other areas (*p* < 0.05). Values of 2.14, 2.33, and 2.65 N · cm/s were obtained for the initial, tapered, and platform areas, respectively. The removal torque for six of the implant designs (Bone Level RC 8, 10, and 12 mm; Tapered Effect RN 10 mm; Brånemark MKIII 10 mm, MKIV 10 mm) was significantly smaller than the corresponding insertion torque (*p* < 0.05). However, the removal torque for ST6, 8, and 10 was almost the same as or slightly greater than the corresponding insertion torque.

**Conclusions:**

The insertion torque-time curves and design features of the implants were accurately transferred. Increasing implant taper angle appeared to increase the torque rate. Torque was mainly generated from the superior surface to the valley of the thread and the inferior and axial surfaces of the platform, while the inferior and axial surfaces of the thread did not significantly affect torque generation.

## Background

Primary stability following implant placement is an essential condition for osseointegration. The primary stability is affected by the implant design, including surface-modifying or implant cavity-forming techniques, as well as by the bone quantity and bone density of the patient [1–3]. In recent years, the interest of implant manufacturers and clinicians has shifted to the acquisition of good fixation, especially for incurable cases in which the density of cancellous bone, such as the maxilla molar part, is low and the cortical bone is thin [4, 5], for which the effects of surface-modifying techniques are low and focus has been changing to the design of the entire implant [6]. Among implant designs, it has been described that good primary stability can be achieved for long and thick implants [7–10] with a small pitch [11] and a taper [6, 12–18], but the cited reports only evaluated primary stability for the entire implant. An implant has characteristic shapes such as parallel, tapered, and platform areas, and the overall design is constructed by placing these areas together. However, there have been no reports of studies that quantitatively measured and evaluated individual designs involved in primary stability. It has been reported that quantitative techniques are necessary to enable the criteria for successful endosseous implants to be more clearly defined [17]. Periotest [19, 20], resonant vibration frequency analysis [21], implant torque value [22], and removal torque value [23] are used as quantitative primary stability evaluations. However, in the periotest and resonant vibration frequency analysis, only general numerical values can be obtained from an implant and it is impossible to perform analyses for individual designs. Furthermore, regarding the implant torque value and removal torque value, analyses for individual designs are impossible as long as only the maximum torque value is used in the conventional methods. Therefore, in this study, for the purpose of measuring the effects of individual implant designs quantitatively, simulation experiments with artificial bone were performed.

## Methods

### Implants

**Fig. 1:**
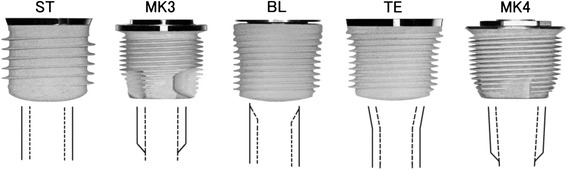
Compressed longitudinally to one third for characteristics of implant design. *ST* Straumann standard implant, *MK3* Nobel Biocare MKIII, *BL* Straumann bone level implant, *TE* Straumann tapered effect implant, *MK4* Nobel Biocare MKIV. Outer surface of implant (*solid line*). Inner surface of implant (*dotted line*)

The type of implant used for the experiments and the characteristics of its design are shown in Table 1 and Fig. 1, respectively. Figure 1 shows that the implant is compressed longitudinally to one third. The outer surface of the implant is indicated with a solid line, and the inner surface of the implant is indicated with a dotted line.

**Table 1:** The type of the implant used for experiment

System	Length	Pitch	Lead	Code	Manufacturer
	(mm)	(mm)	(mm)		
Standard RN	6, 8, 10	1.2	1.2	ST	Straumann
Bone Level RC	8, 10, 12	0.8	0.8	BL	Straumann
Tapered Effect RN	10	0.8	0.8	TE	Straumann
Brånemark MKIII	10	0.6	1.2	MK3	Nobel Biocare
Brånemark MKIV	10	0.6	1.2	MK4	Nobel Biocare

### Preparation of an implant socket in artificial bone

For artificial bone, rigid polyurethane foam (Solid Rigid Polyurethane Foam 20 pcf; Sawbones; Pacific Research Laboratories Inc., USA) measuring 18 × 4 × 13 cm and approaching the maxilla molar part bone density (0.32 g/cc) and physical properties (compressive strength, 8.4 MPa; tensile strength, 5.6 MPa; shear strength, 4.3 MPa; coefficient of elasticity, 284 GPa) [24] was employed. An implant socket was formed by making an interval of more than 2 cm, while avoiding axis wobbling as much as possible, with a drill press (ASD-360; Ashina, Hiroshima, Japan) in the artificial bone.

### Measurement of torque-time curves

For measurement of torque-time curves, a torque measurement system capable of high-speed sampling at 1 sample/ms (PC torque analyzer TRQ-5DRU; Vectrix, Tokyo, Japan) was used. The rotational speed at the time of insertion was 15 rpm, the load was 500 g, and the maximum torque value indicated in the torque-time curve (and following implant torque curve) when inserting the implant (Osseoset 200; Nobel Biocare Japan, Tokyo, Japan) was assumed as the insertion torque value. In addition, the removal torque value (RT) was obtained from the removal torque curve when the implant was reversed immediately after insertion.

### Measurement of the rate of torque rise

The average torque rise rate (N · cm/s) in each region was obtained from the point that the origin and torque rose immediately after implantation, with both ends of the region indicating lines, both ends of the region indicating a quadratic function, and the torque values and implant time of both ends indicating a logarithmic function becoming gradual on the implant torque curve, and mean values and SD were calculated.

### Statistical analysis

It was confirmed that the measurement results for the insertion and removal torque values of each implant were normally distributed, and their significant differences were examined by Student’s *t* test and the Tukey–Kramer method (JMP software; SAS Institute Japan, Tokyo, Japan). The significance level was set at *p* = 0.05.

## Results

### Insertion torque

The insertion torque curve was divided into four regions. The first was the region where the torque rose suddenly immediately after insertion, which was seen in all implant bodies (shown as ① in the figure, and hereinafter called the initial area). The second was the region where the torque rose linearly with a moderate gradient, which was seen in all implant bodies except for Brånemark MKIV (MK4) (shown as ② in the figure, and hereinafter called the parallel area). The third was the region where torque rose suddenly, which was seen in Bone Level RC (BL), Tapered Effect RN (TE), and MK (shown as ③ in the figure, and hereinafter called the tapered area). The fourth was the region where the torque reached a critical point, rose suddenly, and then rose gently, which was seen in Brånemark MKIII (MK3) and MK4 (shown as ④ in the figure, and hereinafter called the tapered area).

**Fig. 2:**
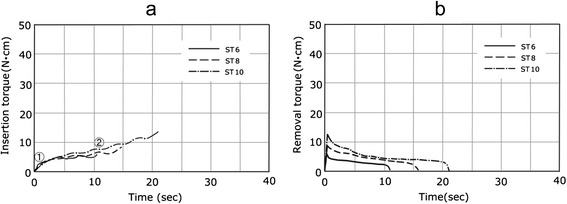
Torque-time curves of the ST. **a** Insertion torque. **b** Removal torque

Regarding the Standard RN (ST), the axial surface and lateral surface were parallel, while for ST6, 8, and 10, only the length was different. The insertion torque curve of the ST3 class shown in Fig. 2a resembled closely, and moderate gradient lines were presented after the initial area in which the torque rose immediately after implantation (parallel area). The length and insertion torque values of the parallel area varied as the length of the implant varied among 6, 8, and 10 mm.

**Fig. 3:**
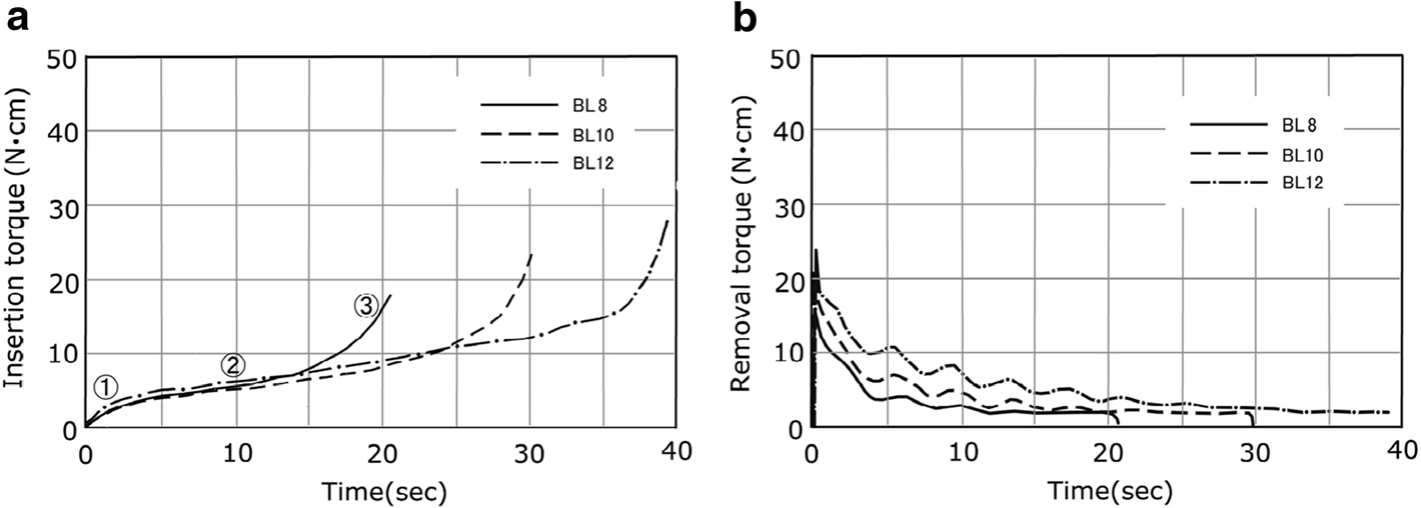
Torque-time curves of the BL. **a** Insertion torque. **b** Removal torque

**Fig. 4:**
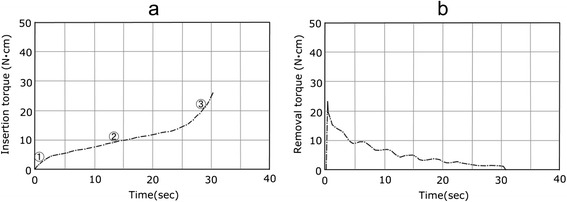
Torque-time curves of the TE. **a** Insertion torque. **b** Removal torque

**Fig. 5:**
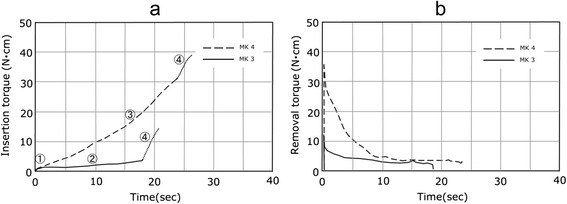
Torque-time curves of the MK3 and MK4. **a** Insertion torque. **b** Removal torque

The torque curve for BL3 in Fig. 3a presented a sudden rise in torque on a quadratic function in the initial area immediately after insertion and a subsequent parallel area (tapered area). As the length of the implant varied among 8, 10, and 12 mm, only the length of the parallel area changed, and the initial area and tapered area had almost the same form. The lateral surface for BL was entirely parallel, while the axial surface had a taper only in the cervical region and the thread of the area was decreased in height. The torque curve for the BL3 class in Fig. 3a showed a rapid rise in torque in the form of a tapered area in the initial area immediately after implantation and in a subsequent parallel area. When the length of the implant varied among 8, 10, and 12 mm, only the length of the parallel area changed, and the initial area and tapered area had almost the same form. For the TE, the lower part of the implant was parallel and the lateral surface and axial surface had the same taper in the cervical region. TE10 in Fig. 4a showed a torque curve with a similar form to BL10 in Fig. 3 and had three kinds of areas. The torque curves for MK3 and MK4 in Fig. 4a had an area in which the torque finally rose suddenly after reaching its critical point and became moderate. This was distinguished from the tapered areas of BL and TE and assumed to be a platform area. MK4 had a gentle taper on the entire axial surface and platform. The platform area, shown in ④, was seen at the end of the torque curve in Fig. 5a, and its torque value was the maximum value among the nine kinds evaluated in this study. In past reports, MK3 was parallel, similar to ST, and had a platform, similar to MK4, and a platform area was seen in the last part of the torque curve. In MK3, the platform area followed the initial area and parallel area, although it presented a final torque value of 4.3 N · cm in the parallel area and then rose further to 10.7 N · cm in the platform area.

### Removal torque

In the removal torque curve, the characteristics of the implant design were not clearly recognized, compared with the implant torque curve. In all implants, the torque rose suddenly immediately after removal was started and reached a peak value. The changes in torque from the peak value were classified into two types. For ST shown in Fig. 2b, the torque fell gently from the peak value. For BL, TE, MK3, and MK4 in Figs. 3b, 4b, and 5b, a sudden fall in torque was seen immediately after the peak value, and it then fell gently. Table 2 shows the maximal values for insertion torque value (IT) and RT obtained from the torque curves and the *p* values obtained by significance tests for RT and IT. The RT for six kinds of implants (BL8, 10, 12, TE10, MK3, MK4) was smaller than the corresponding IT, with statistical significance (*p* < 0.05), while the RT for ST6, 8, and 10 was almost the same as or slightly greater than the corresponding IT.

**Table 2:** Insertion torque value and removal torque value

Code	Insertion torque	Removal torque	Effective thread length (ETL)
	(N · cm)	(N · cm)	(×π mm)
ST6	6.19 ± 0.716	5.95 ± 0.718	11.53
ST8	8.06 ± 1.038	9.09 ± 1.093	15.11
ST10	13.13 ± 1.763	12.37 ± 1.746	21.48
BL8	17.67 ± 1.290	16.67 ± 2.140	20.88
BL10	23.56 ± 1.628	21.99 ± 1.530	31.00
BL12	26.66 ± 3.897	24.40 ± 2.298	39.96
TE10	25.17 ± 2.374	23.76 ± 2.027	31.11
MK3	16.03 ± 0.516	10.30 ± 0.708	39.38
MK4	39.35 ± 0.494	34.31 ± 0502	54.81
			±:SD

### Torque rise rate

Table 3 shows the average torque rise obtained from the torque curve according to areas. The mean torque rise rate of the parallel area was the smallest at 0.36 N · cm/s and differed significantly from those of the other areas (*p* < 0.05). Specifically, the rates were 2.14, 2.33, and 2.65 N · cm/s for the initial area, tapered area, and platform area, respectively, and greater than those of the parallel area by 6–7 times, although significant differences were not recognized among the mean values of these three areas.

**Table 3:** Torque rise rate of the each area (N · cm/s)

	Initial area	Parallel area	Tapered area	Platform area
ST6	1.42 ± 0.43	0.31 ± 0.14	–	–
ST8	3.57 ± 1.62	0.35 ± 0.09	–	–
ST10	2.49 ± 0.81	0.45 ± 0.05	–	–
BL8	2.16 ± 0.21	0.50 ± 0.08	2.32 ± 0.52	–
BL10	1.96 ± 0.49	0.35+0.04	2.45+0.23	–
BL12	1.82 ± 0.27	0.33 ± 0.02	3.41 ± 0.64	–
TE10	2.37 ± 0.42	0.44 ± 0.05	2.30 ± 0.29	–
MK3	2.16 ± 0.28	0.15 ± 0.02	–	3.33 ± 0.85
MK4	2.00 ± 0.00	–	1.30 ± 0.02	2.44 ± 0.09
Average	2.22 ± 0.60	0.36 ± 0.18	2.36 ± 0.75	2.89 ± 0.63
				±:SD

## Discussion

### Insertion torque curve

In 2000, O’Sullivan reported torque curves for a prosthetic implant for the first time, and evaluated the characteristics of the tapered type by torque curves obtained by inserting five kinds of implants into the maxillary bone of unembalmed human cadavers. In a subsequent review, Meredith [25] cited six kinds of torque curves when the final osteotomy diameter was changed and described that a torque curve rose more markedly with a thinner implant cavity. In 2011, Kim et al. [26] compared a case with a self-tapping blade and a case without a self-tapping blade using their torque curves with artificial bone. In 2012, Park et al. [27] obtained the maximum insertion torque, angular momentum, and total insertion energy by torque curves, although quantitative analyses on whether torque curves were correlated with implant designs were not conducted. Furthermore, most conventional studies on torque have focused on measurement [28–31] of the maximum torque value and/or RT at the time of IT or the relationship between the RFA value and IT and/or RT [6, 13, 16, 18, 32]. The present study was not limited to measurements of IT and RT, as the characteristics of the torque curves were divided into four areas designated as the initial, parallel, tapered, and platform areas, and quantitative analyses were performed for each area. For the initial area, a rapid rise in torque occurring immediately after insertion for 1–2 s was observed, and torque value rises of 1.43–2.26 N · cm were also recognized. This reflects torque generated by the implant placed at the predetermined position on the prepared hole rotating and rubbing with the artificial bone with the load of 500 g, and it is presumed that the rapid rise in the initial area was a phenomenon when the thread ridge was inserted into the artificial bone. This indicates that the friction at the time of rotating and pressing is greater than that at the time of rotating and cutting the bones with a tap and is a reasonable result. In the parallel area, the torque curve was a line with a moderate gradient, and the torque rise rate obtained from the gradient of the line was 0.36 N · cm/s. From this, it is estimated that the torque increase when one revolution is added to the parallel thread is about 1.44 N · cm. The preceding thread goes forward and spreads out the bones consistently, and the following thread of the same size does not cause new torque at the time of plastic deformation, and it is therefore presumed that the torque rise in the parallel area is moderate. In the tapered area, the torque curve presented a quadratic curve steadily, the torque rise rate was 2.33 N · cm/s, and the torque increase when one revolution was added was as great as 9.32 N · cm. From this, it is supposed that an increase in the tapered thread is an effective method to increase the torque efficiently. In the tapered area, the diameters of the following threads continued to increase consistently, plastic deformation was caused in all threads in the tapered area, and a torque curve in which the torque continued to increase was produced as a result. It is supposed that the first rapid rise in the platform area was observed when the platform bottom compressed the artificial bone and that stress relaxation of the artificial bone made it more moderate subsequently. The torque rise rate by the platform was 2.65 N · cm/s, which was greater than that of the tapered area.

### Removal torque curve

There have been reports on the removal torque curve of a prosthetic implant. Although the removal torque curves measured in the present study had similar shapes to one another, they were divided into two groups upon detailed observation, comprising a group of ST with parallel only, and a group of BL, TE, MK3, and MK4 having tapers and platforms. Since the thread contacts the artificial bone sequentially at the time of insertion, the torque curves showed the characteristics of each area. At the time of removal, since all threads come in contact with the artificial bone at first, the torque curve did not present the characteristics of the design until it reached the peak value. However, it is estimated that when the thread begins to move subsequently, the difference in design of each area appeared in the torque curve. It seems that the change in torque after reaching the peak value at the time of removal is important information for predicting the influence of the change in primary stability occurring through instant load and the early load on secondary stability. The torque value that instantly decreased with a tapered implant was as small as 4–7 N · cm, and it is necessary to study this further in the future.

### Comparison between IT and RT

The purpose of measuring and evaluating RT in the present study was to clarify whether the implant stability evaluated by IT can be guaranteed even immediately after insertion. In this study, RT was smaller than IT in the implants having tapered and/or platform areas and a significant difference was recognized, while in the design with only a parallel area, no significant difference was seen between IT and RT or RT was slightly greater than IT. Previous studies that measured both IT and RT include those using artificial bone [19, 33], human bone [6, 16, 34], and animal bones [35, 36]. Among such studies, IT and RT were small in those using artificial bone, and RT was smaller than IT. Therefore, using IT to assess the primary stability of an implant revealed the need for certain adjustments.

### Influence of cortical bone

The reason why a simulation test for only cancellous bone without cortical bone was performed in the present study has already been described. It was reported that bone density and the ratio of cortical bone and cancellous bone have influence on the primary stability of an implant and that higher primary stability is achieved with thread, even at the slightest level, binding to cortical bone rather than being surrounded by only cancellous bones [32]. Therefore, it is expected that torque will rise at the end of the torque curve in the cortical bone region and that the torque will further grow by a synergistic effect with factors that increase the torque, such as a taper or platform of an implant. In the simulation experiments in this study, quantitative measurements were successfully performed by extracting only the effects of implant designs and by using a uniform pseudo bone without cortical bones. Sufficient torque is needed for primary stability of an implant, although the risk that excessive compressive force acts on the bone to cause bone resorption and further bone necrosis has been pointed out [37, 38]. To avoid such a situation, it is necessary to find a balance between local bone resorption and the torque, and Meredith [25] recommended insertion torque values of 25–30 N · cm. The torque value and torque rising rate according to the design of implant bodies obtained in the present study enabled estimation of the part of bones generating retention and identification of the part giving compressive force to bones. This will allow us to clarify the relationships among the design of an implant, the value of the torque generated by the implant, and the compressive force to the bones.

## Conclusions

In the torque-duration curve at the time of insertion, the characteristics of the implant design are well shown. It is presented as a straight line with a moderate gradient in the parallel thread area, a quadratic curve-like curve in the tapered area, and a hyperbola-like curve in the platform area. The torque rise rate was 2.14 N · cm/s for the initial area, 0.36 for the parallel area, 2.33 for the tapered area, and 2.65 for the platform area. The torque-duration curves at the time of removal were classified into tapered implants with the peak magnitude as the maximum torque value and straight implants with a maximum torque value greater than the peak magnitude. The RT of the implants having tapered or platform areas was significantly smaller than the corresponding IT, while the RT of the straight implants was the same as or slightly greater than the corresponding IT.

## Abbreviations

BL: Bone Level RC
IT: insertion torque value
MK3: Brånemark MKIII
MK4: Brånemark MKIV
RT: removal torque value
ST: Standard RN
TE: Tapered Effect RN
